# BRAF Inhibitor Resistance in Melanoma: Mechanisms and Alternative Therapeutic Strategies

**DOI:** 10.1007/s11864-022-01006-7

**Published:** 2022-10-01

**Authors:** Jingqin Zhong, Wangjun Yan, Chunmeng Wang, Wanlin Liu, Xinyi Lin, Zijian Zou, Wei Sun, Yong Chen

**Affiliations:** Department of Musculoskeletal Oncology, Fudan University Shanghai Cancer Center, 270 Dongan Road, Xuhui, Shanghai, China

**Keywords:** Melanoma, BRAF mutation, BRAF inhibitor, Targeted therapy, Resistance mechanism, Combination therapy

## Abstract

Melanoma is caused by a variety of somatic mutations, and among these mutations, BRAF mutation occurs most frequently and has routinely been evaluated as a critical diagnostic biomarker in clinical practice. The introduction of targeted agents for BRAF-mutant melanoma has significantly improved overall survival in a large proportion of patients. However, there is BRAF inhibitor resistance in most patients, and its mechanisms are complicated and need further clarification. Additionally, treatment approaches to overcome resistance have evolved rapidly, shifting from monotherapy to multimodality treatment, which has dramatically improved patient outcomes in clinical trials and practice. This review highlights the mechanisms of BRAF inhibitor resistance in melanoma and discusses the current state of its therapeutic approaches that can be further explored in clinical practice.

## Introduction

Melanoma is a tumor caused by malignant transformation of melanocytes and a large variety of somatic mutations. Initiated by receptor tyrosine kinase (RTK) and RAS activation, the RAF-MEK-ERK axis is involved in regulating the main physiological processes, such as proliferation, cell cycle, and apoptosis [1]. Activation of *BRAF*, accounting for 41–55%, is the most common genetic alteration in the occurrence of melanoma [2]. Among BRAF mutations, over 90% are at codon 600, and among these, over 90% are single nucleotide mutations resulting in substitution of glutamic acid for valine (BRAFV600E: GTG>GAG). The second most common mutation is BRAFV600K (GTG>AAG), which represents 5–6%, followed by BRAFV600R (GTG>AGG), BRAFV600'E2' (GTG>GAA), and BRAFV600D (GTG>GAT) [3].

Many BRAF inhibitors (BRAFi) have been developed and approved by the US Food and Drug Administration (FDA), including vemurafenib, dabrafenib, and encorafenib. However, resistance to BRAFi develops quickly, with a median progression-free survival (PFS) of nine months [4]. Thanks to the use of MEK inhibitors (MEKi) including trametinib, binimetinib, and cobimetinib with BRAFi, the metastasis-free survival (MFS) and PFSs for combined therapies increased [5]. Unfortunately, combined agents also develop acquired resistance at a median of 9–11 months [6], which remains a challenge. Therefore, in this review, we will provide deep insight into the BRAF gene to further describe resistance mechanisms and effective strategies for overcoming resistance and to explore potential treatments with the aim of improving outcomes in patients with BRAF-mutations.

## Mechanisms of BRAF inhibitor resistance

**Fig. 1:**
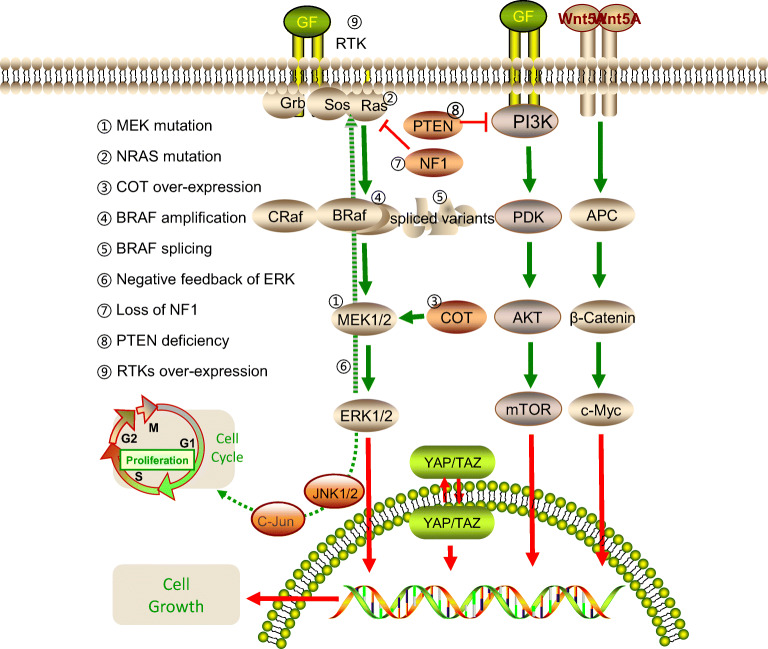
The genetic mechanisms of BRAF inhibitor resistance.

**Fig. 2:**
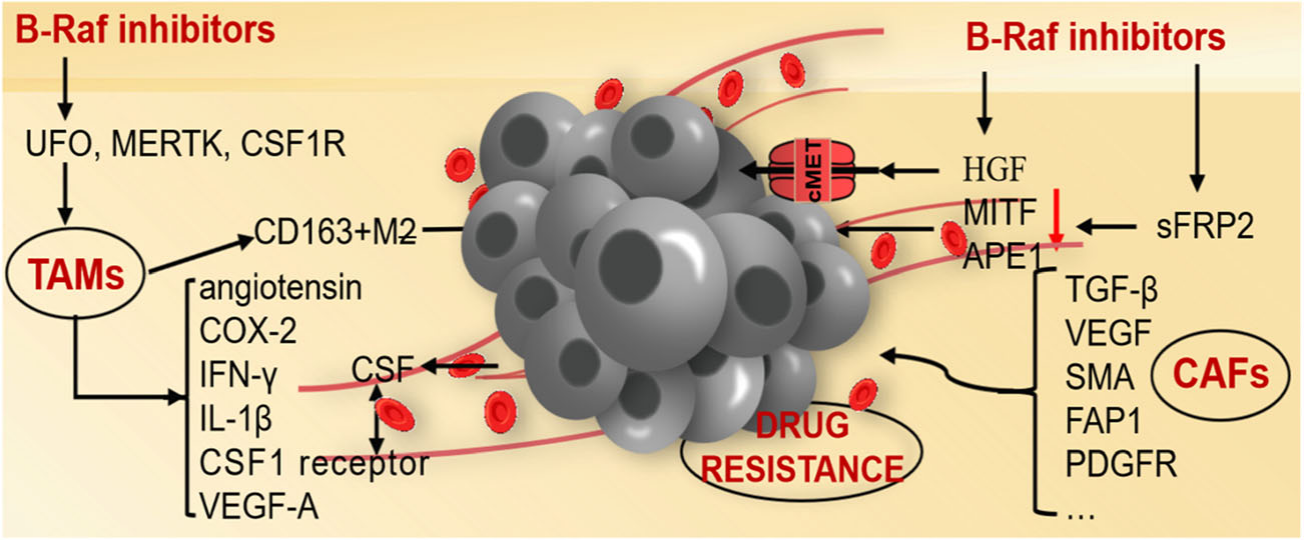
The role of tumor microenvironment in BRAF inhibitor resistance.

Multiple mechanisms have been identified to result in resistance which include genomic (Figure 1) or epigenetic abnormalities and the tumor microenvironment (Figure 2).

### Genetic and epigenetic changes serve critical functions

#### Reactivation of the BRAF/MEK transduction pathway

The recovery of MAPK signaling is the most common mechanism of BRAFi resistance. Deregulation at each point may contribute to resistance [7].

##### RTKs

The hyperactivation of RTKs could promote resistance by activating parallel pathways or directly activating RAS, involving receptors such as PDGFRb, EGFR, MET, KIT, and IGF-1R [8, 9].

##### RAS gene

Constitutive activation of mutated RAS increases BRAF dimerization and subsequent reactivation of MAPK [10]. For example, mutation of NRAS could induce the dimerization of BRAF and CRAF [11].

##### BRAF gene

Overexpression of mutated BRAF, favoring dimerization, results in BRAFi inefficiency and reactivation of the ERK pathway [12]. Moreover, the splicing variant of BRAFV600E, like p61BRAFV600E, can form dimers independently of RAS, making BRAFi ineffective as they only block monomers [13]. Additionally, activation of ARAF and CRAF induces BRAFi resistance, as all RAF isoforms can do the same. Biochemically after treatment with vemurafenib, USP28 expression increased which connects with FBW7 to regulate the stability of RAF and forms a complex to target BRAF for degradation [14].

##### MEK

Mutations in MEK1/MEK2 make BRAFi ineffective regardless of its inhibition as signals below BRAF in the MAPK pathway can still be transduced. The type of MEK1/2 mutations presented varying states of resistance. MEK1 point mutations in C121S, E203K, Q56P, K57E, and MEK2 point mutations, including E207K and Q60P, are associated with BRAFi resistance [15, 16], activating downstream ERK, jumping over BRAF stimulation and leading to resistance. Additionally, overexpression of COT can reactivate MEK, developing resistance to BRAFi [17].

##### ERK

A resetting event of ERK1/2 pathway in adaptive response to BRAFi was proven. Theoretically, the existence of BRAFi is associated with low expression of active RAS, but with the reduction of SPRY2, DUSP, and SPRY expression tested by microarray analysis after vemurafenib, RAS activation reoccurs [18]. Additionally, BRAFi inhibits tumor growth by inhibiting ERK, which in turn inhibits the negative feedback inhibition of ERK on RAS, partially restoring RAS activity and leading to the formation of BRAF dimers induced by RAS [10]. Additionally, the loss of NF1 can result in RAS resistance to negative feedback [19], and NF1 inactivation in melanoma harboring BRAF mutation results in a selective advantage by reversing oncogene-mediated suppression of RAS, which is driven by ERK-induced negative feedback [20].

#### Upregulation of bypass activation

##### The PI3K-AKT-mTOR pathway

Despite continuous targeted inhibition, bypass tracks could eventually lead to the abnormal activation of the downstream pathway. Notably, the more potent target inhibitors are used, the more frequently bypass tracks are likely to be developed [21]. There are four major oncogenic signaling pathways that drive cell growth and proliferation: the PI3K, YAP/TAZ, STAT/JAK, and WNT5A/β-catenin pathways.

The PI3K/AKT/mTOR pathway, which provides antiapoptotic signals and increases proliferation, interacts with MAPK pathways at multiple points. Adaptive PI3K/AKT activity may occur when ERK signaling is blocked, which compensates for BRAFi. The PI3K/AKT pathway is activated by growth factors binding RTKs, so when BRAF is blocked, tumor cells can overexpress RTK, leading to permanent PI3K/AKT signaling [22]. Additionally, PDGFRβ, IGFR1 and EGFR overexpression were reported to cause this pathway reactivation in BRAFi-resistant melanoma [23]. Additionally, mutations in the PI3K/AKT genes induce AKT phosphorylation, which increases anti-apoptotic signaling and the expression of key proliferation genes, independent of BRAF [24]. It is necessary for signal transduction to switch from PIP2 to PIP3, which is promoted by PI3K and negatively regulated by PTEN in PI3K signaling [25], so the activation of AKT after PTEN deficiency is also necessary for intrinsic resistance to BRAFi [26]. These changes make it possible for melanoma cells to proliferate independently of BRAF and are significantly involved in adaptive resistance to BRAFi.

##### YAP/TAZ pathway

Regulated by the homologous proteins Yes-associated protein (YAP) and the transcriptional coactivator with PDZ-binding motif (TAZ), the Hippo pathway is involved in malignant transformation and the growth and metastasis of cancer stem cells. In melanoma cells resistant to BRAFi, the occurrence of nuclear translocation of YAP and TAZ increased and the expression of cell cycle molecules was then promoted [27, 28]. Additionally, the knockdown of YAP or TAZ was reported to suppress the viability of melanoma cells resistant to BRAFi [29]. Therefore, the activation of the YAP/TAZ pathway renders resistance to BRAFi [28], which was proven to be correlated with continuous ERK1/2 activity [27].

##### JNK/c-Jun pathway

The JNK/c-Jun pathway is recognized as an important regulator of cell proliferation, metabolism, and death, and JNKs also belong to the MAPK pathway [30]. C-Jun in melanoma is thought to function downstream of ERK by promoting the transcription of cyclin D1, a positive regulator of the G1-S cell cycle transition. Single-cell analysis suggests that p-c-Jun upregulation contributes to resistance to vemurafenib in some populations by decoupling the inhibition of proliferation from the induction of apoptosis. Additionally, exposing cells to vemurafenib and JNK inhibitors such as JNK-IN-8 results in synergistic cell killing [30, 31].

##### WNT5A/β-catenin pathway

Numerous studies now identify aberrations in β-catenin-independent WNT pathways in melanoma, most notably activated by WNT5A [32]. WNT5A protein and transcript levels were dramatically increased in BRAFi-resistant cells. In vitro studies demonstrated that a loss of WNT5A reduced the viability of cells in the presence of BRAFi. WNT5A-dependent signaling promotes the resistance of melanoma cells to BRAFi via its receptors RYK and FZD7 and the activation of PI3K/AKT signaling has also been reported [33].

#### Epigenetic mechanisms

Epigenetic alterations refer to heritable changes that modulate gene transcription without causing changes in DNA sequences [34]. Epigenetic mechanisms regarding BRAFi resistance include DNA methylation, noncoding RNAs, histone-modifying enzymes, and histone modifications.

##### DNA methylation

Hypomethylation, defined as the absence of methyl groups from cytosines, can open tightly packed chromatin, leading to genomic instability. In contrast, hypermethylation can lead to transcriptional repression [35]. Drug resistance corresponding to transcriptomic and methylomic alterations was identified [36] and transcriptomic analysis revealed differential mRNA expression in genes connected with differential methylation at CpG clusters and short DNA sequences that are mainly CG-rich and remain unmethylated [37], suggesting key connections between drug resistance and epigenetic regulation of DNA methylation.

Mutations in DNA methyltransferases (DNMTs), such as DNMT3B, considered a de novo DNA methyltransferase for depositing and maintaining methyl marks [38], have a role in tumor progression. Low global DNA methylation levels appeared in drug-tolerant melanoma cells following targeted treatment as DNMT3A, DNMT3B, and DNMT1 were differentially expressed [39].

##### Histone-modifying enzymes (HMEs) and posttranslational modifications (PTMs)

The N-terminal tails of histone proteins forming a histone octamer can be covalently and reversibly changed with various kinds of PTMs. These dynamic histone PTMs and specific histone proteins can promote either transcriptional activation or repression of targeted genes by remodeling their chromatin structures [36].

The expression of histone demethylases like KDM6A, KDM6B, KDM1B, JARID1A, and JARID1B is elevated in melanoma with a drug-tolerant state, which is accompanied by increased levels of H3K9me3 and lower levels of H3K4me3 and H3K27me3, indicating selected gene silencing and epigenetic activation. SETDB1 and SETDB2, histone methyltransferases, are also upregulated after treatment with BRAFi and MEKi and their knockdown restored drug sensitivity [39]. In addition, the histone deacetylase SIRT6 was downregulated in BRAFi-resistant melanoma cells, leading to upregulation of the IGF-1 receptor (IGF-1R) and subsequent AKT pathway activation [40].

##### Noncoding RNAs

Previous studies have identified miRNAs and lncRNAs as effectors of resistance to BRAFi (Table 1).

**Table 1:** Non-coding RNAs associated with BRAFi resistance in melanomas

Types of ncRNA	Name	Expression after resistance	Mechanisms in BRAFi resistance	Ref
miRNA	miR-514a	↑	Decreased expression of the tumor suppressor NF1	[41]
	miR-125a	↑	Inhibiting pro-apoptotic parts of intrinsic apoptosis pathway	[42]
	miR-211-5p	↑	Increased expression of MITF regulating TRPM1 gene resulting in activation of the survival pathway	[43]
	miR-34a, miR-100, and miR-125b	↑	Involvement in the control of cell proliferation and apoptosis	[44]
	miR-204-5p and miR-211-5p	↑	Stimulation in Ras and MAPK upregulation	[45]
	miRNA-204 and miRNA-211	↑	Reducing expression of NUAK1/ARK5 proteins	[46]
	miR-1246	↑	G2/M arrest and autophagy	[47]
	miR-7	↓	Increased expressions of EGFR, IGF-1R, and CRAF and further activation of MAPK and PI3K/AKT pathway	[48]
	miR-579-3p	↓	Negative correlation with BRAF in resistant cells	[49]
	miR-200c	↓	Deactivation of the PI3K/AKT and MAPK signaling cascades, and acquisition of epithelial-mesenchymal transition-like phenotypes	[50]
lncRNA	EMICERI	↑	Regulating MOB3B whose over-expression downregulates LATS1 to activate the Hippo signaling pathway	[51]
	MIRAT	↑	Binding to IQGAP1 and facilitating signaling through the MAPK pathway	[52]
	TUG1	↑	Acting as an oncogene sponging miR-129-5p and inducing cell growth and invasion	[46]
	TSLNC8	↑	Binding with the catalytic sub-unit of PP1α to regulate its distribution, and re-activating the MAPK signaling	[53]
	SAMMSON	↑	Interacting with p32 and regulating the metabolism of mitochondria and CARF-p53 signaling pathway	[54] [55]
	RMEL3	Unknown	A positive regulator of PI3K and MAPK signaling in melanoma	[56]
	IGF2AS, MEG3, and Zeb2NAT	↑	Might serve as prognostic markers of response to vemurafenib treatment in melanoma patients	[57]

In detail, it contains information regarding the expression levels after BRAFi resistance (↑or↓), the mechanisms in BRAFi resistance the references in which they are described.

### Influences of the tumor microenvironment

Resistance to BRAFi in melanoma is known to develop not only as a result of genomic or epigenetic abnormalities, but the role of tumor microenvironment is also important. Recent research has identified the role of intratumoral fibroblasts and macrophages in the development of resistance.

#### Cancer-associated fibroblasts (CAFs)

CAFs, differing from normal fibroblasts by upregulated expression of vimentin, fibroblast activation protein-1 (FAP1), and α-smooth-muscle actin (SMA), as well as PDGFR and TGFβ signaling [58], have been reported to allow therapeutic escape from BRAFi.

In the neighborhood of CAFs, melanoma cells present an aggressive and dedifferentiated mesenchymal phenotype. After treatment with BRAFi, melanoma cells maintain high levels of active mTOR signaling, facilitating protein synthesis, cell growth, and utilization of nutrients from the microenvironment [59]. They also respond to growth factors and cytokines secreted by CAFs, including TGF-β and VEGF, which promote cell survival and growth [58]. Vemurafenib directly activates fibroblasts to secrete hepatocyte growth factor (HGF), which activates both the MAPK/ERK and PI3K/AKT signaling pathways and downregulates the expression of proapoptotic genes [60]. It is interesting to discover that aging fibroblasts related to aged melanoma are more invasive and were shown to secrete frizzled related protein 2 (sFRP2), inhibiting β-catenin and downregulate expression of MITF and apurinic endonuclease (APE1), rendering cells resistant to BRAFi [61].

CAFs also secrete extracellular matrix (ECM) components. For example, ECM-induced integrin signaling promotes resistance to BRAFi. In regard to interaction, BRAFi-resistant melanoma cells release TGF-β, promoting CAF differentiation, which in turn increases the expression of ECM molecules and further develops to BRAFi resistance [62].

#### Tumor-associated macrophages (TAMs)

TAMs, which are activated M2 macrophages, express various anti-inflammatory factors and an immune-suppressive microenvironment was built. A high number of intratumoral CD163+ macrophages correlate with BRAFi resistance. The transition from macrophages to CD163+ M2 macrophages is induced by exosome-derived growth factors and interleukins released by melanoma cells, T-regulatory cells, and other macrophages. In the paracrine mode, macrophages that express the colony-stimulating factor 1 receptor (CSF1R) respond to CSF secreted by melanoma cells, which also stimulates resistance in autocrine manners [58]. In turn, TAMs secrete the melanoma-stimulating molecules angiotensin, COX-2, IFN-γ, and IL-1β, supporting melanoma growth and metastasis [63].

Moreover, BRAFi itself also stimulates TAMs. For instance, it induces the production of VEGF-A, which stimulates not only angiogenesis in the tumor but also macrophage survival and tumor immune escape [64]. Additionally, TAMs secrete TNFα, which promotes MITF expression and inhibits BRAF protein to block apoptosis in melanoma [65]. For vemurafenib, TAMs protected melanoma cells from BRAFi-induced apoptosis but did not disturb the G2/M phase [66].

## Current treatment strategies to overcome BRAFi resistance

One current strategy to prevent or delay resistance is to develop combination therapies. A complementary strategy is to develop novel MAPKi to tackle resistance mechanisms and offer new options for future therapeutic development.

### Development of combination therapies

Current clinical trials of combination therapies to overcome BRAF inhibitor resistance in patients with melanoma are listed in Table 2.

**Table 2:** Current clinical trials of combination therapies to overcome the BRAF inhibitors resistance in patients with melanoma

TrialID	Public title	Year	Recruitment Status	Phase	Intervention	Drug target
NCT04903119	Nilotinib Plus Dabrafenib/Trametinib in Metastatic Melanoma	2021	Recruiting	1	Trametinib, Dabrafenib, Nilotinib	MEK, BRAF, KIT, PDGFR
NCT04557956	Testing the Addition of the Anti-cancer Drug, Tazemetostat, to the Usual Treatment (Dabrafenib and Trametinib) for Metastatic Melanoma That Has Progressed on the Usual Treatment	2020	Recruiting	1/2	Trametinib, Dabrafenib, Tazemetostat	MEK, BRAF, EZH2
NCT04527549	Testing Dabrafenib and Trametinib With or Without Hydroxychloroquine in Stage IIIC or IV BRAF V600E/K Melanoma	2020	Recruiting	2	Trametinib, Dabrafenib, Hydroxychloroquine	MEK, BRAF, Autophagy
NCT04375527	Binimetinib and Nivolumab for the Treatment of Locally Advanced Unresectable or Metastatic BRAF V600 Wildtype Melanoma	2020	Recruiting	2	Binimetinib, Nivolumab	MEK, PD-1
NCT03972046	Neoadjuvant Use of Talimogene Laherparepvec and BRAF/MEK Inhibitor for Advanced Nodal BRAF Mutant Melanoma	2019	Active, not recruiting	2	Trametinib, Dabrafenib, Talimogene laherparepvec (T-Vec)	MEK, BRAF, GM-CSF
NCT04201457	A Trial of Dabrafenib, Trametinib and Hydroxychloroquine for Patients With Recurrent LGG or HGG With a BRAF Aberration	2019	Recruiting	1/2	Trametinib, Dabrafenib, Hydroxychloroquine	MEK, BRAF, Autophagy
NCT03580382	Study of CDX-3379, a Human Monoclonal Antibody Targeting ERBB3, in Combination With the MEK Inhibitor, Trametinib, in Patients With Advanced Stage NRAS Mutant and BRAF/NRAS Wildtype (WT) Melanoma	2018	Active, not recruiting	1/2	Trametinib, CDX-3379	MEK, ERBB3
NCT03543969	Adaptive BRAF-MEK Inhibitor Therapy for Advanced BRAF Mutant Melanoma	2018	Recruiting	1	Cobimetinib, Vemurafenib	MEK, BRAF
NCT03455764	MCS110 With BRAF/MEK Inhibition in Patients With Melanoma	2018	Active, not recruiting	1/2	Trametinib, Dabrafenib, MCS110	MEK, BRAF, CSF-1
NCT03668431	Dabrafenib + Trametinib + PDR001 In Colorectal Cancer	2018	Recruiting	2	Trametinib, Dabrafenib, PDR001	MEK, BRAF, PD-1
NCT03693170	Encorafenib, Binimetinib and Cetuximab in Subjects With Previously Untreated BRAF-mutant ColoRectal Cancer ANCHOR-CRC	2018	Active, not recruiting	2	Binimetinib, encorafenib, Cetuximab	MEK, BRAF, EGFR
NCT03101254	LY3022855 With BRAF/MEK Inhibition in Patients With Melanoma	2017	Active, not recruiting	1/2	Cobimetinib, Vemurafenib, LY3022855	MEK, BRAF, CSF-1
NCT03026517	Clinical Trial of Phenformin in Combination With BRAF Inhibitor + MEK Inhibitor for Patients With BRAF-mutated Melanoma	2017	Recruiting	1	Trametinib, Dabrafenib, Phenformin	MEK, BRAF, Activation of AMPK
NCT03272464	INCB039110 in Combination With Dabrafenib and Trametinib in Patients With BRAFmutant Melanoma and Other Solid Tumors.	2017	Active, not recruiting	1	Trametinib, Dabrafenib, INCB039110	MEK, BRAF, JAK1
NCT02967692	A Study of the Anti-PD1 Antibody PDR001, in Combination With Dabrafenib and Trametinib in Advanced Melanoma	2016	Active, not recruiting	3	Trametinib, Dabrafenib, Spartalizumab	MEK, BRAF, PD-1
NCT02858921	Neoadjuvant Dabrafenib, Trametinib and/or Pembrolizumab in BRAF Mutant Resectable Stage III Melanoma Neo Trio	2016	Active, not recruiting	2	Trametinib, Dabrafenib, Pembrolizumab	MEK, BRAF, PD-1
NCT02382549	A Clinical Trial to Evaluate a Melanoma Helper Peptide Vaccine Plus Dabrafenib and Trametinib	2015	Recruiting	1	Trametinib, Dabrafenib, 6MHP	MEK, BRAF, Induce T cell and Ab responses

#### Combination of BRAF and MEK inhibitors

Combining BRAFi with MEKi delayed the emergence of resistance to a single BRAFi and significantly inhibited melanoma growth [67], which also reduced the occurrence of proliferative skin lesions and secondary malignancies [68].

Findings from clinical trials confirmed that combination treatment (dabrafenib/trametinib and vemurafenib/cobimetinib) had higher response rates (approximately 65–70%), improved progression-free survival (median, approximately 12 months), and improved overall survival (median, approximately 24 months) with less cutaneous toxicity, and such combinations are now the standard targeted therapy for patients with BRAF-mutant melanoma.

#### Combination of PI3K/AKT inhibitors and MAPK inhibitors

The sensitivity of BRAF-mutant melanoma to BRAFi or MEKi can be enhanced through combined inhibition of the PI3K/AKT pathway, which has synergistic effects in inducing apoptosis [69]. In a study using uveal melanomas harboring activated Q209 L/P mutations, a combination of MEKi GSK1120212 and pan-PI3K inhibitor GSK2126458 induced significant cell apoptosis in comparison with inhibition of either pathway alone [70].

Aside from boosting the efficacy of MAPK inhibitors, addition of PI3K/AKT inhibitors may overcome their resistance. A study showed that the phosphorylation of AKT and MAPK downstream target S6 did not decrease in resistant melanoma, which means that an increased AKT pathway may contribute to resistance. In support of this, the combination of BRAFi and MEKi with an AKT inhibitor reversed the resistance of melanoma cells to vemurafenib [71]. Similarly, the addition of the PI3K inhibitor GSK2118136 to either dabrafenib or trametinib also delayed resistance [67].

As BRAF-mutant melanoma resistant to treatment with BRAFi or MEKi showed suppression of mTOR activity [72], targeting downstream effector mTOR was also included to disrupt aberrant PI3K/AKT signaling. Strangely, the inhibition of BRAF, PI3K, and mTOR suppressed melanoma cell growth but was ineffective at inducing cell death, while a combination of PI3K and mTOR inhibitors with a MEKi induced apoptosis [9].

Despite promising preclinical data, the clinical combination of BRAFi or MEKi with PI3K/AKT inhibitors was disappointing. A phase 1 trial (NCT01476137) testing the safety of trametinib in combination with the AKT inhibitor afuresertib showed only a partial response (PR). Other clinical trials are currently undergoing, such as a combination of the PI3K inhibitor copanlisib with a MEKi in melanoma (NCT01392521). However, due to their early-phase nature, it is not possible to determine the efficacy of these combinations until initiation of phase 2 and 3 trials with larger patient cohorts [73].

#### Combination of BRAF and/or MEK inhibitors with immunotherapy

Immune checkpoint inhibitors (ICIs), involving inhibitors targeting cytotoxic T lymphocyte antigen 4 (CTLA-4) and programmed death 1 (PD-1) receptors (i.e., ipilimumab, nivolumab, and pembrolizumab), have lower response rates but more durable responses than BRAFi. Preclinical and translational data indicate that combining BRAFi and MEKi with ICI could exceed the limitations of each class and potentially lead to longer-lasting responses [74••].

Combined treatment with vemurafenib and adoptive lymphocyte transfer therapy in vivo showed better antitumor effects than either treatment alone [75]. Mechanistically, treatment with a BRAF inhibitor alone or in combination with a MEK inhibitor is associated with increased CD8 + T-cell infiltration, increased T-cell cytotoxicity, and decreased expression of immunosuppressive molecules such as interleukins-6 and interleukins-8, which further supports the potential of combining BRAF/MEK inhibitors with immunotherapy [76].

PD-L1, an immunosuppressive ligand of PD-1 receptors, is also associated with PI3K-mediated resistance to the BRAFi vemurafenib [77], and its upregulation in tumor cells that have acquired resistance to BRAFi has been reported [78]. Hence, concurrent inhibition of the MAPK pathway and immune checkpoint signaling may prove a potent antitumor combination therapy, which means that the combination of trametinib with immune checkpoint inhibitors targeting PD-1, PD-L1, or CTLA-4 was more effective than use of each inhibitor alone.

Clinical trials combining MAPK dual-targeted inhibitors with immune checkpoint inhibitors for melanoma are currently underway (NCT01940809, NCT02130466, and NCT02858921) [79, 80]. Among the results of Keynote-022, IMspire 150, and COMBI-i, COMBI-i achieved the highest response rate of 78%, but there was no significant difference in the objective response rate (ORR) between the triple-drug group and dual-target group in all three studies, indicating that the dual-target ORR reached the ideal effective rate. In addition, according to the COMBI-i study, triple-drug combination may be more applicable to patients with a larger tumor burden [LDH ≥ 2 ULN, number of metastases ≥ 3, sum of lesion diameters ≥ 66 mm], and higher tumor mutation load (TMB) ≥ 10 mt/M. However, complementarily, with the comparison of these three clinical studies, the incidence of adverse reactions of the triplet regimens was relatively high, with an incidence of adverse reactions of grade 3 and above of more than 70%, and the IMspire150 study even reached nearly 80%. Therefore, we can conclude that targeted combined immunization can be better applied and promoted if the adverse reaction rates can be effectively controlled.

#### Combination with synergistic small molecules

Several synergistic small molecules have emerged to restore the efficacy of vemurafenib sensitivity. HSP90 inhibition may be a highly effective strategy for managing vemurafenib resistance [81]. Fluvastatin, the BET-bromodomain inhibitor JQ1, and the natural product neferine have been shown to reduce vemurafenib resistance in melanoma [82]. CHMFL-BMX-078, the BMX inhibitor, significantly decreased the phosphorylation of AKT, and had high selectivity for vemurafenib-resistant melanoma cells. Combination treatment with vemurafenib and CHMFL-BMX-078 inhibited AKT pathway and ERK signaling pathway, which explains their enhanced inhibitory effects. Furthermore, the aptamer LL4A selectively inhibits vemurafenib-resistant melanoma by binding to the CD63 protein [83]. However, there are currently no viable methods for entirely reversing vemurafenib resistance, and the development of novel combinations is still urgently needed.

### Strategies for blocking MAPK signaling

#### Inhibition of mutant RAF signaling

Two different strategies are currently under investigation for the inhibition of mutant RAF and mitigation of paradoxical activation — pan-RAF inhibitors and paradox breakers.

Type II pan-RAF inhibitors target both active RAF dimers and monomers and therefore inhibit ERK signaling including AZ628, CCT241161, and TAK-580 [84]. However, their uses in vivo have been more limited because of their lack of selectivity for mutant BRAF, in contrast with vemurafenib and dabrafenib, which could lead to greater toxicity and disruption of WT RAF signaling [85].

A third generation of RAF inhibitors, known as “paradox breakers,” inhibits BRAF without promoting dimerization, thereby preventing paradoxical upregulation of ERK signaling [86]. Paradox breakers have the potential to be effective against V600E mutation splice variants and upstream RAS mutations and are currently under development [87]. PLX8394, a BRAF-specific dimer breaker, selectively disrupts BRAF homo and BRAF-CRAF heterodimers, which will be effective for treatment of tumors with class 1 or 2 BRAF mutants, BRAF fusions and RAS-independent, BRAF dimer-dependent resistance to current BRAFi [88]. Unfortunately, this drug has been shown to paradoxically activate RAF signaling, and BRAF fusions could drive acquired resistance to PLX8394 noted in some preclinical trials [89].

#### Vertical blockade of MAPK signaling

Adequate adaptive barriers can be ensured in vertical blockade of MAPK signaling through polytherapy to suppress BRAFi resistance. Interestingly, preclinical modeling suggests that intermittent BRAF inhibitor therapy may delay resistance [90]. However, S1320, a phase 2 clinical trial evaluating whether intermittent dosing of dabrafenib and trametinib improves PFS in patients with metastatic and unresectable BRAFV600 melanoma, demonstrates that continuous dosing yields superior PFS compared to intermittent dosing in patients with BRAFV600E and BRAFV600K melanoma [91]. A similar vertical blockade strategy was adopted in a phase II clinical trial of metastatic colon cancer where EGFR, BRAF, and MEK were coinhibited. The results indicated not only a superior ORR but also PFS and OS, as described above [92, 93]. Moreover, recent studies have shown that SHP2 inhibitors are effective in bypassing the paradoxical activation of MAPK signaling upon BRAF inhibition in RAS-mutant cancers [94]. These studies highlight the importance of molecular classification and profiling to better predict therapy regimens.

## Potential and future direction

In view of the complex drug resistance mechanism, individualized and precise management should be an inevitable choice in clinical practice. However, at the same time, as researchers, there are still many issues that need to be explored. The recently resolved BRAF-MEK1-14-3-3 complexes uncovered molecular details of how BRAF is regulated by phosphorylation and protein-protein interactions [95], which may provide information for the assembly of complexes targeting MAPK signaling pathways. These interactions may serve as good candidates for combination approaches to provide synergistic effects and reduce possible side effects. Moreover, with a handful of FDA-approved RAF/MEK inhibitors widely used in the clinic, the impact of inhibiting RAF and/or MEK on somatic tissue and tumor-infiltrating immune cells has recently been brought into the spotlight. Another challenge is to understand the dimerization mechanism which will enable further improvement of the current inhibitors in designing tailored drugs. Deeper investigation into these issues will lead to the development of new strategies to achieve therapeutic goals.

New innovative therapies are also needed to address drug resistance. Proteolysis targeting chimeras (PROTACs), a novel strategy to knock down proteins of interest [96], have potential advantages over conventional small molecule inhibitors [97]. To address the limitations of BRAFi therapies, a study [98••] using vemurafenib-based PROTACs achieved low nanomolar degradation of all classes of BRAF mutants but WT RAF and outperformed vemurafenib in inhibiting cancer cell growth. Additionally, the modular design of PROTACs makes it ideal for providing tailored drugs and personalized medicine. On the other hand, the discovery of the key mechanisms regulating TME, it may have a beneficial antitumor effect. At the same time, attention should be paid to the relationship between changes in tumor cell intrinsic signaling pathways and T-cell rejection or infiltration. The development of new targeted therapy based on TME can also be a new therapeutic strategy.
