# Correction to: BRAF Inhibitor Resistance in Meslanoma: Mechanisms and Alternative Therapeutic Strategies

**DOI:** 10.1007/s11864-022-01038-z

**Published:** 2022-12-02

**Authors:** Jingqin Zhong, Wangjun Yan, Chunmeng Wang, Wanlin Liu, Xinyi Lin, Zijian Zou, Wei Sun, Yong Chen

**Affiliations:** Department of Musculoskeletal Oncology, Fudan University Shanghai Cancer Center, 270 Dongan Road, Xuhui, Shanghai, China

**Correction to: Current Treatment Options in Oncology (2022) 23:1503-1521**

10.1007/s11864-022-01006-7

The original version of this article, unfortunately, contained mistakes.

The author list was arranged incorrectly. The correct sequence of authors is presented below:

Jingqin Zhong, MD

Wangjun Yan, PhD

Chunmeng Wang, PhD

Wanlin Liu, MD

Xinyi Lin, MD

Zijian Zou, MD

Wei Sun, PhD*

Yong Chen, PhD*

*Email: Yong Chen, chenyong@fudan.edu.cn; Wei Sun, wsun14@fudan.edu.cn

